# Supplementary material for: Self-propagating wave drives morphogenesis of skull bones in vivo
Source: Nat Commun. 2025 May 9;16:4330. doi: 10.1038/s41467-025-59164-9 (PMC12064835; doi:10.1038/s41467-025-59164-9)
Supplement: Supplementary file 1 — Supplementary Information [file 41467_2025_59164_MOESM1_ESM.pdf]

Supplementary Figures

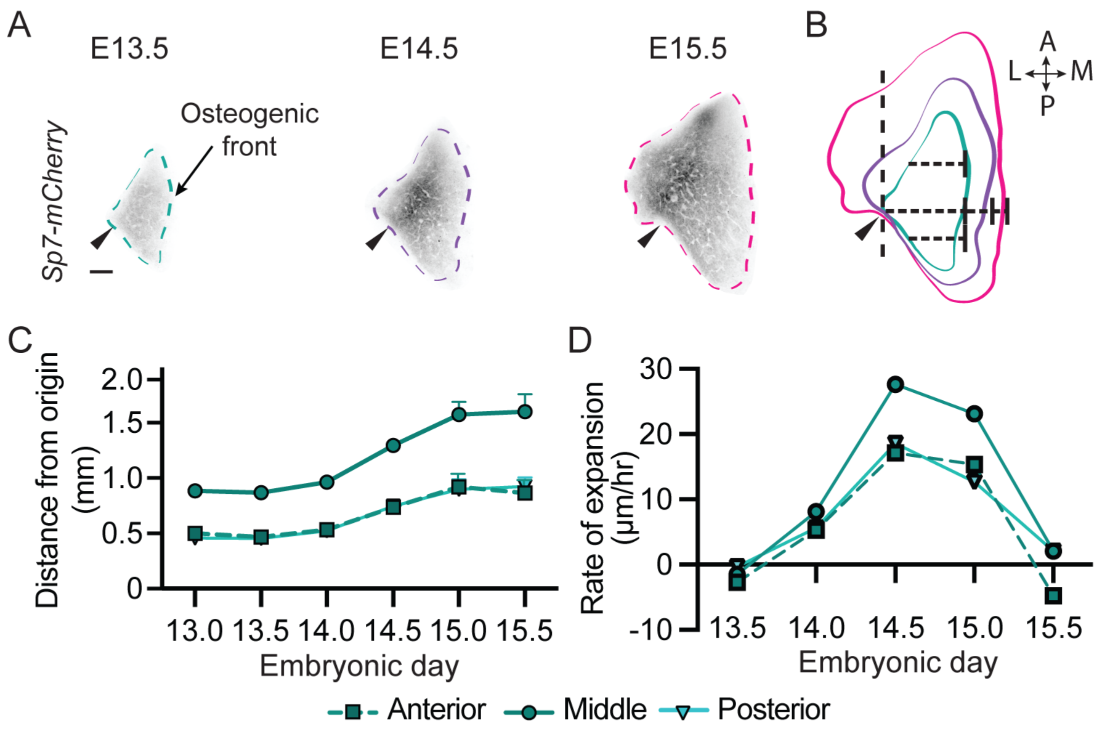

Figure S1: Growth rates of *ex vivo* skull cap explants and *in vivo* skull caps are comparable. **(A)** Frontal bones cropped from flat-mounted skull caps at E13.5, E14.5 and E15.5. Arrows indicate landmarks used to align bones from different samples to one another. **(B)** Example of overlaid bone outlines from different stages. Size measurements were performed at anterior, middle and posterior locations of the bones. A, anterior; P, posterior; L, lateral; M, medial. Scale bar = $500\,\mu\mathrm{m}$. **(C)** Quantification of medial bone extension between E13.0 and E15.5 at the anterior, middle and posterior positions as indicated in **(C)**. Error bars indicate SD. **(D)** Rates of expansion obtained by comparing subsequent time points for the measurements shown in **(C)**.

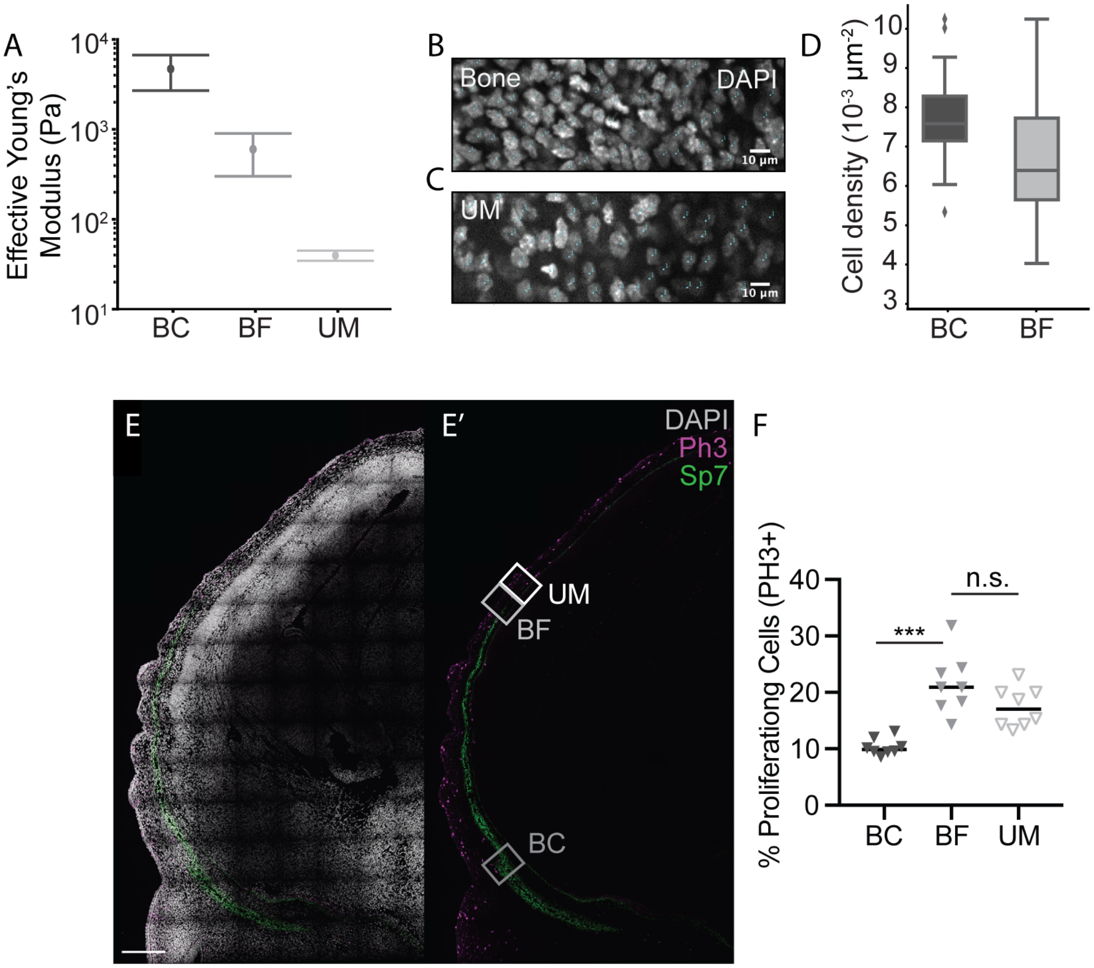

Figure S2: Developing skull bones show regional differences in stiffness, cell density and cell proliferation rates. **(A)** NanoIndenter measurements of the stiffness (apparent Young's modulus) of E14.5 mouse embryos at the Bone Center (BC), Bone Front (BF) and Undifferentiated Mesenchyme (UM) (indicated in Fig. 1B). **(B-C)** Representative DAPI images showing cell nuclei in the bone **(B)** and the undifferentiated mesenchyme **(C)** for an E14.0 control embryo. **(D)** Quantification of cell densities in the bone (n=22) and the mesenchyme (n=22) for E14 embryos (N=4 for BAPN, N=11 for Control). Two-sided Mann-Whitney U test, $p = 0.00812$. **(E-E')** representative confocal image of coronal sections through the mouse E14.5 head labelled with DAPI (white), Ph3 (magenta), and Sp7 (Green) showing where ROIs were selected for quantifying the percentage of Ph3 positive mesenchyme in the Undifferentiated mesenchyme (UM), bone front (BF), and bone center (BC). **(F)** Proliferation rates of E14.5 embryos quantified by Ph3+ staining at the Bone Center (N=8, n=360), Bone Front (N=8, n=110) and Undifferentiated Mesenchyme (N=8, n=1177) of the tissue (Mann-Whitney test, $p > 0.05$). Solid lines show the mean.

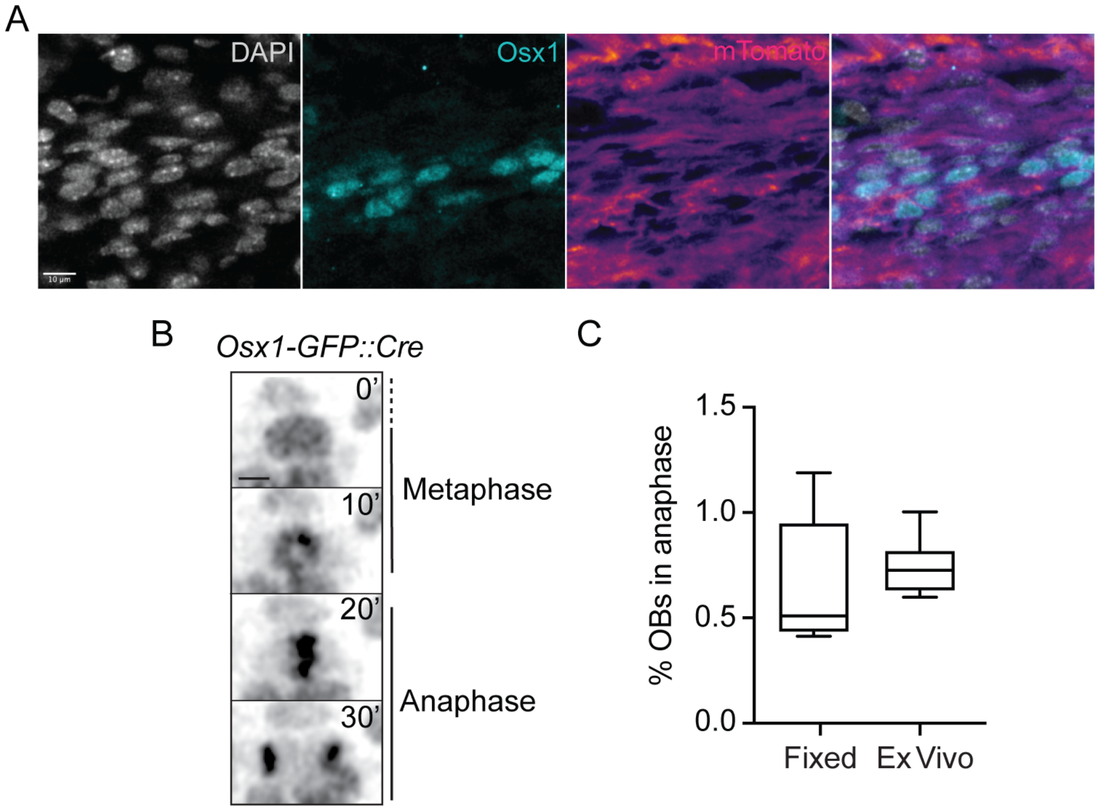

Figure S3: Supplemental Figure S3. Cell division rates in *ex vivo* experiments at E14.5 match *ex vivo* rates. **(A)** Stainings of cell nucleus (DAPI), osteoblasts (Green) and cell membranes (Tomato). We use the *R26R-mTmG* in the absence of a Cre driver to observe cell membranes together with an Osx1 antibody to mark the osteoblasts. **(B)** Stills separated by $10\,\mathrm{min}$ of a single *Osx1-GFP::Cre* labelled nucleus during metaphase and anaphase. **(C)** Fraction of osteoblasts in anaphase in fixed tissue images compared to *ex vivo* images.

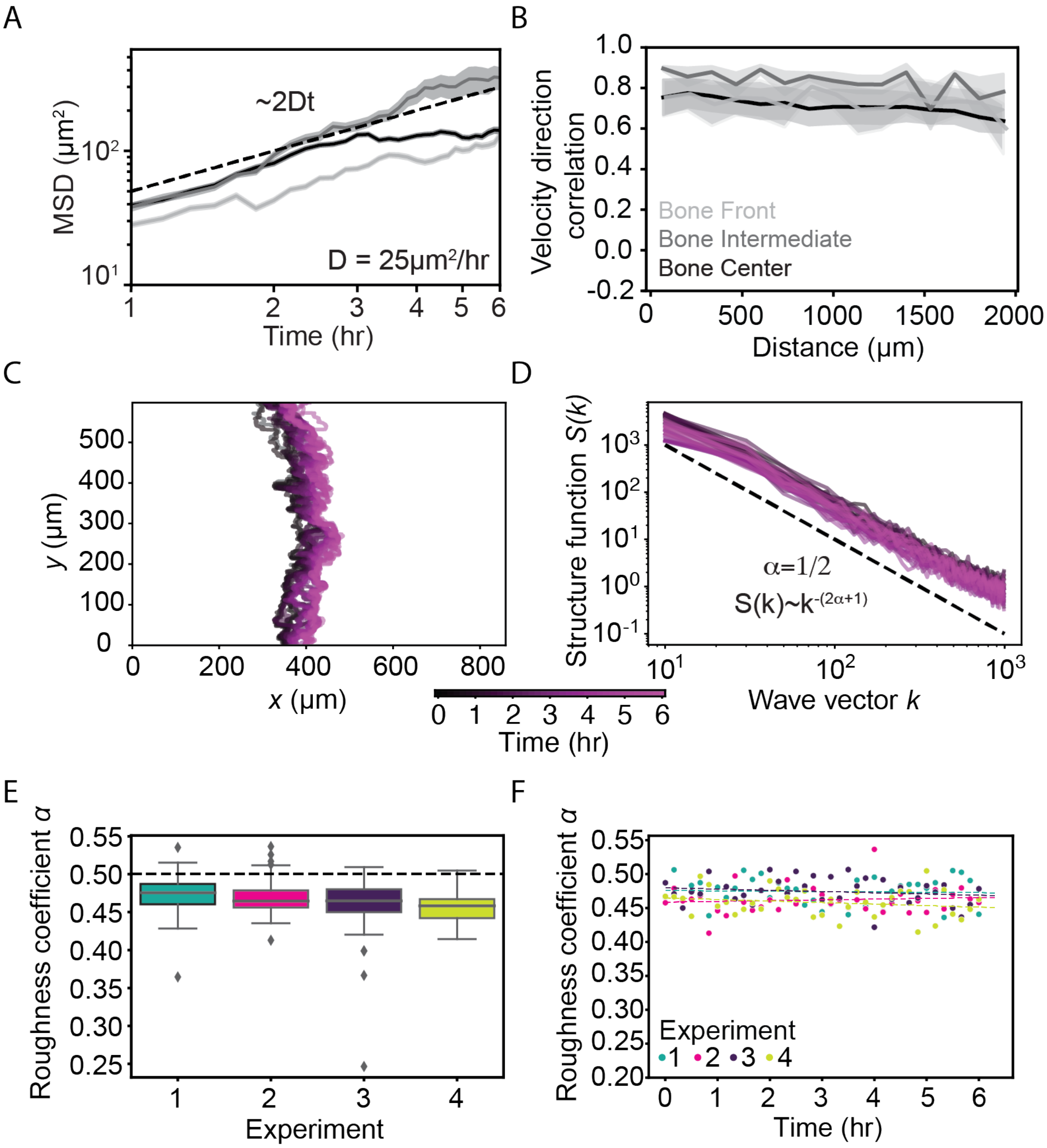

Figure S4: Further quantifications of cell tracks and osteogenic front. **(A)** Mean-squared displacements of the relative displacement tracks in Fig. 1H. Shaded areas show SEM. The dotted line represents diffusive motion with a diffusion constant of $D = 25\,\mu\text{m}^2/\text{h}$. **(B)** Normalised velocity correlation at $2\,\text{h}$ for four individual movies. Lateral, intermediate and front osteoblast tracks are labelled in light grey, grey and black, respectively. Shading indicated SEM. **(C)** Osteogenic fronts for different time frames of a representative live imaging movie (Supplemental Movie 2). **(D)** The structure function for the osteogenic fronts shown in **(C)** together scales as a power law, the slope of which defines the roughness coefficient of the interfaces. The dashed line corresponds to a roughness coefficient of 1/2. **(E)** Box plots for roughness coefficients of all frames for four *ex vivo* live imaging experiments. The dotted line marks the value of 1/2. **(F)** Roughness coefficients across time for four *ex vivo* live imaging experiments together with linear fits (dotted lines).

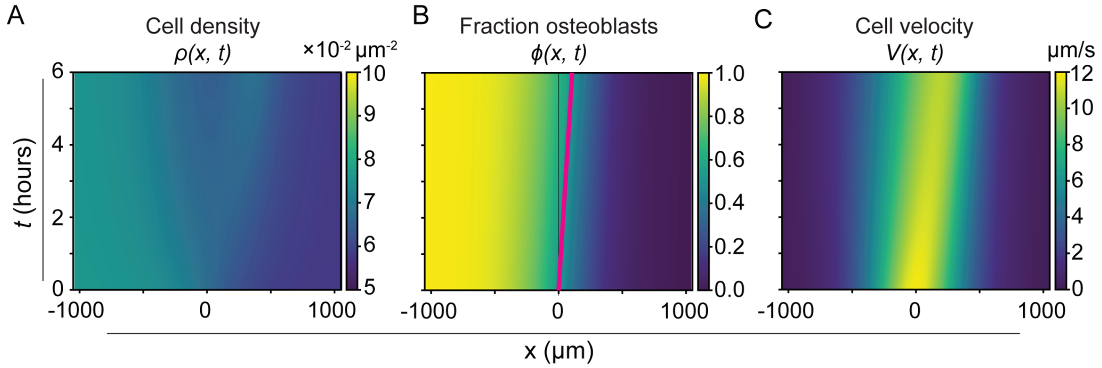

Figure S5: Theoretical model simulations. **(A-C)** Kymographs showing the dynamics of the simulated theoretical model with realistic parameters (see Table S1) for the **(A)** cell density, **(B)** fraction of osteoblasts and **(C)** cell velocity. The magenta line in B indicates a fixed position on the wavefront.

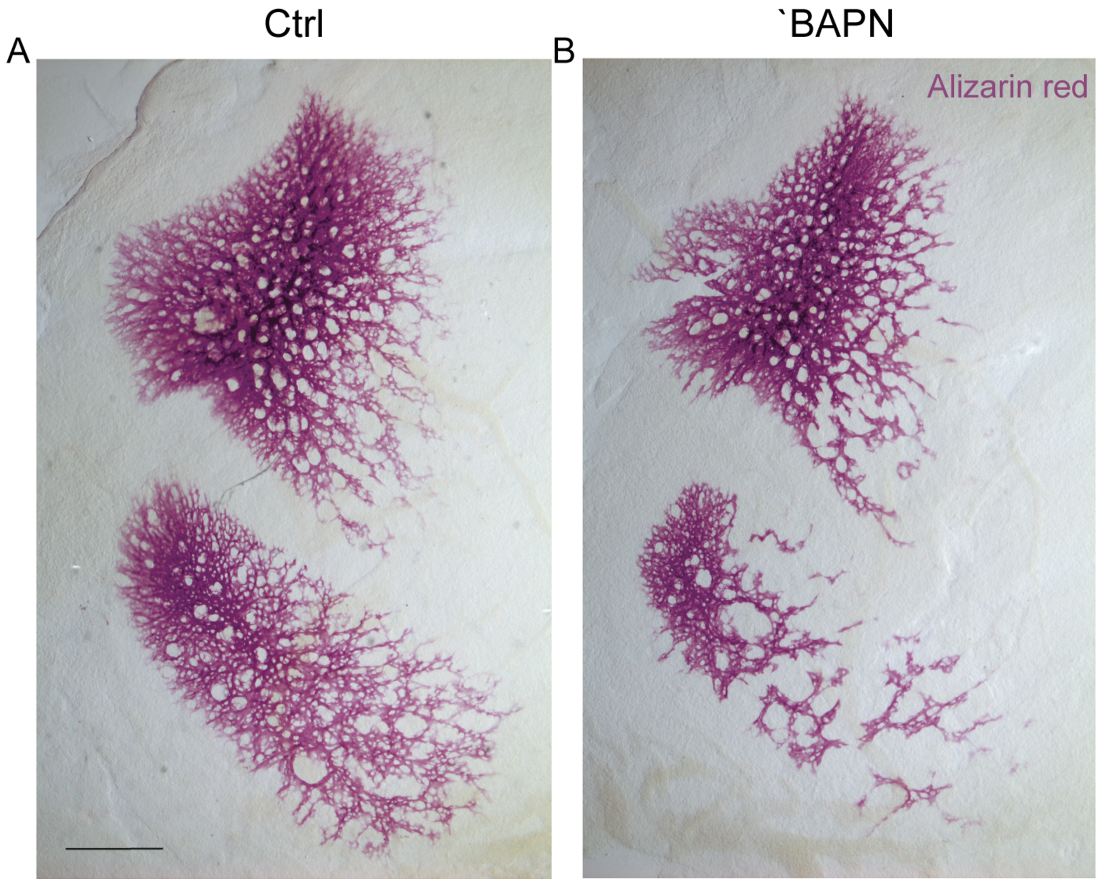

Figure S6: BAPN treatment alters mineralised bone **(A-B)** Flatmounted Alizarin red stained skull cap from E15.5 control (A) and BAPN-fed (B) embryos (n = 7, N = 2). Scale bar = 500 μm.

Theoretical model

We constructed a continuum model for a tissue with two cell types, described in terms of spatially and temporally varying fields of cell density, cell flows and mechanical stresses. Let $\rho_i(x,t)$ $(i = A, B)$ be the concentrations of cells of type A and B. Although the model formulation is generic, in the context of our system we take A to be the undifferentiated mesenchymal progenitors and B the differentiated osteoblast population. Consider two different processes that can change these concentrations:

1. Proliferation and cell death give rise to effective reproduction rates $k_i(\rho_i)$ $(i = A, B)$, which depend on local cell densities as described below in Eq. 8.

2. A is converted to B irreversibly at a rate k_D, which can depend on other (dynamic) variables of the system, as will be discussed in the next section.

Number balance imposes the following dynamic equations on the cell densities ρ_A, ρ_B:

$$\begin{aligned}
\partial_t \rho_A + \nabla \cdot (\rho_A \mathbf{v_A}) &= k_A\, \rho_A - k_D\, \rho_A \\
\partial_t \rho_B + \nabla \cdot (\rho_B \mathbf{v_B}) &= k_B \rho_B + k_D \rho_A
\end{aligned} \tag{1}$$

These equations describe how the local density of A and B cells can change through advection (with velocities $\mathbf{v_A}, \mathbf{v_B}$), cell division or loss, as well as conversion of A cells into B cells.

As we are interested in the differentiation dynamics of osteoblasts, we make the following transformation (*1*):

$$\rho \equiv \rho_A + \rho_B \tag{2}$$

$$\phi \equiv \frac{\rho_B}{\rho_A + \rho_B} \tag{3}$$

$$\mathbf{v} \equiv (1-\phi)\mathbf{v_A} + \phi \mathbf{v_B} \tag{4}$$

$$\mathbf{J} \equiv \phi(\mathbf{v_B} - \mathbf{v}) \tag{5}$$

Hence, rather than considering individual densities ρ_A, ρ_B, we describe the system in terms of the total concentration of cells ρ and the local fraction of B cells ϕ. Similarly, rather than considering individual cell velocities $\mathbf{v_A}, \mathbf{v_B}$, we consider the $\mathbf{v}, \mathbf{J}$. If we assume that the mass per cell of both cell types is roughly equal, then $\mathbf{v}$ represents the center of mass velocity and $\mathbf{J}$ represents the relative flux of B cells with respect to the center of mass.

From Eq. 1, we then derive the following equations for $\rho(x,t)$ and $\phi(x,t)$:

$$\partial_t \rho + \nabla \cdot (\rho \mathbf{v}) = \left(k_A(1-\phi) + k_B \phi\right) \rho \tag{6}$$

$$\partial_t \phi + \mathbf{v} \cdot \nabla \phi = -\frac{1}{\rho} \nabla \cdot (\rho \mathbf{J}) + (k_B - k_A)\, \phi(1-\phi) + k_D(1-\phi) \tag{7}$$

We assume $\mathbf{J} = -D\nabla\phi$, in analogy to Fick's law for diffusion, but with respect to the relative fraction of B cells ϕ rather than total concentration. This approach is based on the assumption

that in the absence of active processes (proliferation, differentiation), in the long-time limit the system becomes well-mixed, i.e. spatial inhomogeneities in cell numbers of A and B cells vanish and becomes spatially uniform. Provided there is no cell sorting (e.g. due to differential adhesion), random motion of cells would be sufficient to lead to mixing of different cell types. Hence we assume that in the absence of other processes, local differences in cell type composition will eventually relax to a homogeneous state where the local composition is uniform across space.

We impose constraints on tissue growth by assuming that the cell density essentially follows a logistic growth model, such that the net growth rate decreases when the density becomes too high (*2, 3*). Therefore, we write the net division rate as

$$k_i(\rho) = \frac{1}{\tau} \frac{\rho_i^c - \rho}{\rho_i^c}, \tag{8}$$

where ρ_i^c with $i = A, B$ are the carrying capacities for the two cell types and τ sets the time scale of adapting to homeostasis.

To solve the above system of PDEs, we need an additional equation for the advection velocity $\mathbf{v}$, which we derive from force balance. Various studies have argued that the material properties of tissues largely follow that of a viscoelastic medium, with the property that at sufficiently long timescales we can ignore elastic terms and the tissue effectively behaves as a viscous fluid (*3–6*). If we assume the medium is isotropic, the stress tensor in the tissue can be decomposed as (*7*)

$$\begin{aligned} \sigma &= -P\mathbf{I} + \tilde{\sigma}, \\ \tilde{\sigma} &= 2\tilde{\eta}\tilde{\mathbf{v}} + \xi(\nabla \cdot \mathbf{v})\mathbf{I}, \end{aligned} \tag{9}$$

where P is the pressure, $\mathbf{I}$ is the identity matrix, $\tilde{\eta}$ is the dynamic viscosity, ξ is the bulk viscosity, $\tilde{\sigma}$ is the deviatoric stress tensor and its traceless part $\tilde{\mathbf{v}}$ is defined as

$$\tilde{v}_{ij} = \frac{1}{2}\left(\partial_i v_j + \partial_j v_i - \frac{2}{3}(\partial_k v_k)\delta_{ij}\right). \tag{10}$$

Force balance in the tissue then takes the general form

$$\nabla \cdot \sigma = \gamma \mathbf{v} + \mathbf{F}, \tag{11}$$

where γ is the friction coefficient and $\mathbf{F}$ represents external forces acting on the tissue.

Since the tissue we study consists of a thin sheet of cells, we will describe it as an effective 2D system and neglect the z dimension by averaging all quantities in this direction. Furthermore, as our data shows that cells persistently move unidirectionally and that this directionality is correlated over large distances in the direction perpendicular to that of cell motion (Fig.

S4A), so to first approximation we can set $v_y = 0$ and $\partial_y v_x = 0$, such that we obtain an effective one-dimensional description of our system. In particular, using Eqs. 9 and 11 and writing $\eta \equiv \left(\frac{4}{3}\tilde{\eta} + \xi\right)$ and in the absence of external forces ($\mathbf{F} = 0$) we now obtain

$$\eta\, \partial_x^2 v_x - \partial_x P = \gamma v_x \tag{12}$$

To close the system and solve for v, we require an equation of state of the form $P(\rho)$. For tissues, the following form derived from thermodynamics has been proposed (*8, 9*):

$$P(\rho) = E \log(\rho/\rho_0). \tag{13}$$

Here E is the Young's modulus, which in general can be spatially dependent (as will be discussed below), and ρ_0 sets the density at which the pressure is defined to be zero.

Full system

Putting together Eqs. 6, 7, 12 and 13, we obtain the following system of PDEs:

$$\partial_t \rho + \nabla \cdot (\rho \mathbf{v}) = (k_A(1-\phi) + k_B\, \phi)\, \rho \tag{14}$$

$$\partial_t \phi + \mathbf{v} \cdot \nabla \phi = D \frac{1}{\rho} \nabla \cdot (\rho \nabla \phi) + (k_B - k_A)\, \phi(1-\phi) + k_D(1-\phi) \tag{15}$$

$$\nabla \cdot \sigma = \gamma \mathbf{v} \tag{16}$$

$$\sigma = -P\mathbf{I} + 2\tilde{\eta}\tilde{\mathbf{v}} + \xi(\nabla \cdot \mathbf{v})\mathbf{I}, \tag{17}$$

After reduction to 1D and writing $v = v_x$, we obtain

$$\partial_t \rho + \partial_x(\rho v) = (k_A(1-\phi) + k_B\phi)\, \rho \tag{18}$$

$$\partial_t \phi + v\partial_x \phi = D\rho^{-1}\partial_x\left(\rho \partial_x \phi\right) + (k_B - k_A)\, \phi(1-\phi) + k_D(1-\phi) \tag{19}$$

$$\eta \partial_x^2 v - \gamma v = \partial_x P \tag{20}$$

To recapitulate, in the above equation D is the diffusion constant, η is the viscosity, γ is an effective friction term, k_D is the differentiation rate, P is the pressure defined in 13, E is the stiffness and ρ_h is a homeostatic density. The first equation represents cell number balance due to net proliferation of both cell types as well as advective flows. The second equation describes how the osteoblast fraction ϕ changes due to advection, diffusion, cell proliferation, or cell differentiation. Finally, the third equation describes force balance and arises from a balance of viscous stresses with friction and isotropic forces arising from pressure gradients.

We study our system on a bounded spatial domain $x \in [-L, L]$ and take as initial conditions sigmoidal profiles for ρ and ϕ, $\rho_0(x) \equiv \rho(x, 0) = \rho_A^c + (\rho_B^c - \rho_A^c)\frac{1}{1+e^{x/\epsilon}}$ and $\phi_0(x) \equiv \phi(x, 0) = \frac{1}{1+e^{x/\epsilon}}$, where ϵ tunes the sharpness of the profile. As the equation for v has no time derivative, we obtain $v(x, 0)$ as the solution to the non-time dependent Eq. 20 at $t = 0$.

We take Dirichlet boundary conditions for all variables. First, we assume that the cell densities approach their homeostatic values at the boundaries, i.e. $\rho(-L,0) = \rho_0(-L) \approx \rho_B^c$, $\rho(L,0) = \rho_0(L) \approx \rho_A^c$. Furthermore, we assume that all cells at the lateral end (where the bone resides) to have differentiated, while none of the cells at the medial end are differentiated, i.e. $\phi(-L,0) = \phi_0(-L) \approx 1$ and $\phi(L,0) \equiv \phi_0(L) \approx 0$. Finally, we assume that there is no flow of cells at the extreme ends of our system, i.e. $v(-L,0) = v(L,0) = 0$. This assumption holds only at short time scales, as the tissue is spatially confined, while on longer time scales the model would need to be extended to take into account the overall growth of the tissue.

We numerically solve this system using a finite difference scheme. Specifically, we use the explicit method for time-stepping and took centered differences for the spatial derivates. We iteratively solve this system in two steps: first we solve the time-independent PDE for $v(x,t)$ for known profiles of $\rho(x,t)$ and $\phi(x,t)$ at a specific time t and then we solve for $\rho(x,t+1)$ and $\phi(x,t+1)$ using the explicit scheme.

Mechanical feedback

To capture the interaction between material properties and cellular processes in the above system, we introduced a feedback loop in the model that couples these two features. First, we modelled the stiffness to depend locally on the fraction of differentiated osteoblasts through

$$E = (E_A + (E_B - E_A)\phi)\,, \tag{21}$$

where E_A, E_B represent the stiffnesses generated by populations consisting of only mesenchymal and osteoblast cells respectively. This reflects the fact that osteoblasts mineralise the ECM in their immediate vicinity and generate a stiffer environment than mesenchyme progenitors, as shown in our stiffness measurements.

Second, we let the differentiation rate $k_D = k_D(E)$ depend on the local stiffness E, in accordance with *in vitro* experiments establishing a clear role for substrate stiffness in regulating osteoblast differentiation (*10,11,11–17*). In particular, stretch and compression of tissues and/or substrate have been shown to increase osteoblast differentiation and it has been suggested that this is mediated through activation of BMP signaling, ERK signaling and p38 MAPK phosphorylation (*18*). Although the exact quantitative relation between stiffness and differentiation is not known *in vivo*, we next derive a few constraints on this relation.

Wave solutions

We next sought to derive solutions of our model consistent with the observed osteoblast expansion in the system. As the displacement of the osteogenic front increases quasi-monotonically (Fig. 2B) and the GFP reporter intensity profiles have similar shapes across time (Fig. 2D), to first approximation we can model the expansion as a linearly expanding wavefront in osteoblast

density (ϕ). Such a linearly expanding wavefront is consistent with a Fisher-Kolmogorov-Petrovsky-Piskunov (FKPP) wave (*19, 20*), for which the mathematical properties have been extensively studied (*21*). Two observations further demonstrate consistency with an FKPP wave:

1. Direct observation of differentiation events ahead of the osteogenic front in the form of isolated osteoblasts with only nuclear staining and no membrane staining (Fig. 2E-G). This is consistent with a picture where the mesenchymal progenitor state is unstable and fluctuations may induce cell differentiation into osteoblasts.

2. Measurement of the roughness coefficient of the osteogenic front, which agrees with that of the FKPP wave, as shown in Fig. S4D.

We next identify mechanisms of generating a FKPP wave in our system. First note that Eq. 18 in the absence of density gradients ($\partial_x \rho = 0$) and cell movement ($v = 0$) reduces to a generalized FKPP equation of the form

$$\begin{aligned} \partial_t \phi &= D \partial_x^2 \phi + f[\phi], \\ f[\phi] &= (k_B - k_A)\, \phi(1-\phi) + k_D(1-\phi). \end{aligned} \tag{22}$$

A FKPP wave exists if and only if $f[\phi = 0] = f[\phi = 1] = 0$, while $f'[\phi = 0] > 0$ and $f'[\phi = 1] < 0$ (*21*). We first note that in the case $k_D = 0$, this reduces to the original FKPP equation and these conditions are all satisfied provided $k_B - k_A > 0$. This scenario, which has been previously studied in (*1*), when translated to our system would imply that the osteoblasts would divide more than the mesenchymal progenitors and generate a pressure gradient that pushes progenitor cells towards the midline. However, our PH3 immunostaining revealed no significant difference between the bone front and undifferentiated mesenchyme in terms of proliferation rates (Fig. S2E), and therefore we consider $k_A - k_B \approx 0$ in the following.

While $f[\phi = 1] = 0$ is satisfied by definition, the condition $f[\phi = 0] = 0$ becomes a condition on k_D, which depends on the stiffness E and subsequently through Eq. 21 on ϕ in a yet unspecified way. More precisely, $f[\phi = 0] = 0$ implies

$$k_D[E[\phi = 0]] = k_D[E_A] = 0. \tag{23}$$

In other words, the differentiation rate should be zero when the stiffness of the system is that of mesenchymal cells, i.e. in the absence of osteoblasts.

The derivative of $f[\phi]$ takes the form

$$f'[\phi] = -k_D[\phi] + k_D'[\phi](1-\phi), \tag{24}$$

where the apostrophe denotes derivative with respect to ϕ. The conditions on the derivatives then imply

$$f'[\phi = 0] = -k_D[\phi = 0] + k_D'[\phi = 0] = k_D'[\phi = 0] > 0, \tag{25}$$

$$f'[\phi = 1] = -k_D[\phi = 1]) < 0 \Rightarrow k_D[\phi = 1]) > 0. \tag{26}$$

The first condition together with above Eq. 23 imply that while in a system with no osteoblasts ($\phi = 0$) and at the mesenchymal stiffness E_A the differentiation rate k_D should be zero, any slight increase would tilt the differentiation rate constant toward positive values and induce osteoblast differentiation. The second condition is for the differentiation rate at $\phi = 1$, which is in itself irrelevant when all cells have already differentiated. However, assuming continuity of $k_D[\phi]$ would imply that in the vicinity of $\phi = 1$, the differentiation rate remains positive. This would mean that in a field of mostly osteoblasts with a few remaining mesenchymal cells, those remaining cells would differentiate into osteoblasts as well.

In the absence of further information about k_D, we assume a simple linear relation between k_D and stiffness which satisfies the constraints for a FPKK wave, Eqs. 23 and 26 :

$$k_D = \alpha\left(E[\phi] - E_A\right). \tag{27}$$

Here, α represents a proportionality constant quantifying how sensitive the differentiation rate is to change in stiffness. Since we assume that stiffness $E[\phi]$ cannot be lower than the mesenchymal stiffness E_A, for any $\alpha > 0$ we obtain a positive differentiation rate $k_D > 0$. Together with Eq. 21, this choice of k_D (Eq. 27) implies that the total differentiation rate takes the form corresponding exactly to the classical FKPP equation

$$k_D(1-\phi) = \alpha(E_B - E_A)\phi(1-\phi). \tag{28}$$

In this picture, the differentiation rate constant k_D increases monotonically with stiffness, but the differentiation rate $k_D(1-\phi)$ shows a single maximum at $\phi = 1/2$. This is due to the depletion of progenitor cells as ϕ and thereby the stiffness increases, which therefore reduce differentiation until all of the cells are differentiated.

Wave velocities

The velocity of a generalized Fisher's equation of Eq. 22 has the form $v_{FKPP} = 2\sqrt{Df'[\phi]}$. Applied to our system and assuming $\partial_x \rho \approx 0$, we obtain the general solution (not assuming $k_A = k_B$)

$$v_{FKPP} = \sqrt{D((k_B - k_A) + k'_D[\phi] - k_D)}. \tag{29}$$

For the specific choice $k_D = \alpha(E[\phi] - E_A)$ (Eq. 27), we obtain

$$v_{FKPP} = \sqrt{D((k_B - k_A) + \alpha(E_B - E_A))}. \tag{30}$$

In addition to the Fisher velocity, the cells experience an advection velocity derived from the solution to the force balance equation in Eq. 20. For the cells at the front, this can be written as $v_A = v(x = 0, t)$. The front velocity is then to first approximation given by

$$v_F \approx v_{FKPP} + v_A. \tag{31}$$

Thus, we now see two main features contributing to the expansion speed: (1) differences in proliferation rate between the cell types and (2) the stiffness gradient across the tissue. First, the FKPP velocity is minimal when the proliferation rates $k_A = k_B$ and increases as the proliferation rate gradient is increases. Secondly, both the advection velocity as well as the FKPP wave front velocity increases with larger differences in stiffness across the system (i.e. upon increasing $E_B - E_A$). In the case of the advection velocity v_A, this is due to the mechanical effect of increased pressure gradient across the tissue. In the case of the FKPP velocity v_{FKPP}, this arises from an increase of the differentiation rate with larger stiffness differences as described in Eq. 30.

Parameter estimates

We estimated the parameters of the model using different methods: inference from experiment data, literature estimates and parameter fitting (S1). First, we estimated the carrying capacities of both cell types ρ^c_A, ρ^c_B from cell density estimates, which we obtained through manual counting of cells in fixed tissue images (Fig. S2D). We estimated the diffusion constant D from the cell tracks for each of the three groups of tracked cells by taking the residues obtained from subtracting the average motion of all cells in a group (Fig. S2C). The mean squared displacement of these residues scales roughly linearly with time, which in the case of Brownian motion has a slope of $2D$. Next, we estimated the stiffnesses E_A, E_B from the E14.5 NanoIndenter measurements of the apparent Young's Modulus (Fig. S2A).

We estimated τ, which represents the time scale on which division and apoptosis events lead to homeostasis, to be in the order of 10 hrs. The viscosity η and friction coefficient γ could also not be directly estimated from our experimental data, but literature estimations are available for both. Moreover, in the inviscid limit ($\eta \to 0$), the cell velocity v becomes inversely proportional to γ (Eq. 20), such that we can tune this parameter to match the observed cell velocity v_A close to the front. Likewise, we use the measured front velocity v_F to estimate α, which from Eq. 31 can be approximated in the following way that depends only on v_F and parameters known from other estimations:

$$\alpha = \left((v_F - v_A)^2 - D(k_A - k_B)\right) / (E_B - E_A). \tag{32}$$

In conclusion, we have two free parameters γ and α which we fit to the measured velocities v_A and v_F respectively. Thus, the model predictions are in fact the spatial profiles of ρ, ϕ, V that can be compared directly to experimental data.

Analysis of the osteogenic front

Definition of the osteogenic front

We start from maximum projections of the live imaging setup for the *Osx1-GFP::Cre* intensity images, and define coordinates x running along the medial-lateral axis and y in the perpendicular direction. We seek to define an interface that runs vertically along the y axis. We set

Symbol	Interpretation	Estimate	Source
ρ_A^c	Carrying capacity A cells (mesenchymal progenitors)	$6.7 \cdot 10^{-3}\, \mu m^{-2}$	Fixed tissues imaging (Fig. S2D)
ρ_B^c	Carrying capacity B cells (osteoblasts)	$7.7 \cdot 10^{-3}\, \mu m^{-2}$	Fixed tissues imaging (Fig. S2D)
D	Diffusion constant	$15\, \mu m^2/hr$	Live imaging cell tracks (Fig. S2B)
E_A	Stiffness cell type A (mesenchymal progenitors)	30 Pa	Nanoindenter measurements (Fig. S2A)
E_B	Stiffness cell type B (osteoblasts)	1000 Pa	Nanoindenter measurements (Fig. S2A)
τ	Timescale in Eq. 8	10 hr	Estimate from (*9*)
η	Viscosity	$10^4\, Pa \cdot s$	Typically $10^3 - 10^5\, Pa \cdot s$ (*5, 22, 23*)
γ	Friction coefficient	0.8×10^3 Pa	Fit cell velocity; typically $10^3 - 10^5\, \frac{Pa \cdot s}{\mu m^2}$ ((*23, 24*))
α	Coefficient in Eq. 27	3.5×10^{-6}	Fit front velocity v_F using Eq. 32

Table S1: Estimation of model parameters from experiments, literature and parameter fitting.

an automated intensity threshold I using the Otsu method ((*25*)) and segment the image into connected regions where all pixels have intensity values $\geq I$. Because of the overall intensity gradient in the x direction, there typically is a single largest contiguous region of which the boundary spans the entire vertical axis. Note that in general this boundary could curve in such a way that for some position y, there could be multiple points lying on the boundary. Therefore, we first perform an "overhang correction", by taking the value of x that maximizes the size of the contiguous region. The result is then an interface height function $h(y, t)$ for each frame at time t.

Quantification of front roughness

There are different ways of defining the roughness of an interface. Here we choose to define roughness through a method commonly used to characterise interfaces in kinetic growth processes (*26, 27*). Specifically, given an interface height function $h(y, t)$ defined for $y \in \{y_1, \ldots, y_n\}$, we first take the (discrete) Fourier transform $h(k, t) = \sum_{m=0}^{n-1} h(y_m, t) e^{-2\pi i \frac{km}{n}}$. The structure function $S(k, t)$ is then defined as

$$S(k, t) = \langle h(k, t) h(-k, t) \rangle$$

where the average is over replicates of the data (e.g. multiple experiments). Under the scaling hypothesis in interface growth theory, the structure function then scales as (*27*):

$$S(k, t) = k^{-(2\alpha+1)} \tilde{s}(k t^{1/z}),$$

where the dynamic exponent z is a constant and

$$\tilde{s}(u) = \begin{cases} u^{-(2\alpha+1)} & \text{if } u \ll 1 \\ \text{const.} & \text{if } u \gg 1 \end{cases}.$$

Hence, for sufficiently large times t, $S(k,t)$ scales as a power law and we obtain the roughness coefficient α by fitting our interface function to this power law. Note that in practice, fluctuations at very large k (i.e. very small length scales) may deviate from this scaling law. However, these represent fluctuations at spatial scales smaller than that of a single cell. Therefore, in practise we set a cutoff in the wave vector by considering only $k < k_{max} = L/a$ where L is the length of the interface and a is the typical size of a cell ($10\,\mu\text{m}$).

References

1. J. Ranft, M. Aliee, J. Prost, F. Jülicher, J.-F. Joanny, *New Journal of Physics* **16**, 035002 (2014).

2. B. I. Shraiman, *Proceedings of the National Academy of Sciences* **102**, 3318 (2005).

3. M. Basan, T. Risler, J. Joanny, X. Sastre-Garau, J. Prost, *HFSP Journal* **3**, 265 (2009).

4. R. A. Foty, G. Forgacs, C. M. Pfleger, M. S. Steinberg, *Physical Review Letters* **72**, 2298 (1994).

5. G. Forgacs, R. A. Foty, Y. Shafrir, M. S. Steinberg, *Biophysical Journal* **74**, 2227 (1998).

6. J. Ranft, *et al.*, *Proceedings of the National Academy of Sciences* **107**, 20863 (2010).

7. E. Landau, L.D.; Lifschitz, *Course of Theoretical Physics: Vol. 6, Fluid Mechanics*, Course of Theoretical Physics (Pergamon, 1987).

8. J. C. Arciero, Q. Mi, M. F. Branca, D. J. Hackam, D. Swigon, *Biophysical Journal* **100**, 535 (2011).

9. P. Recho, J. Ranft, P. Marcq, *Soft Matter* **12**, 2381 (2016).

10. A. J. Engler, S. Sen, H. L. Sweeney, D. E. Discher, *Cell* **126**, 677 (2006).

11. J. Swift, *et al.*, *Science* **341** (2013).

12. W. J. Hadden, Y. S. Choi, *Journal of biological engineering* **10**, 1 (2016).

13. C. A. Chacón-Martínez, J. Koester, S. A. Wickström, *Development* **145** (2018). Dev165399.

14. J. Lee, A. A. Abdeen, T. H. Huang, K. A. Kilian, *Journal of the mechanical behavior of biomedical materials* **38**, 209 (2014).

15. L. Yuge, *et al.*, *Biochemical and biophysical research communications* **311**, 32 (2003).

16. A. Ignatius, *et al.*, *Biomaterials* **26**, 311 (2005).

17. C. Jacobs, *et al.*, *Journal of biomechanics* **31**, 969 (1998).

18. A. Rutkovskiy, K.-O. Stensløkken, I. Jarle, *Medical Science Monitor Basic Research* **22**, 95 (2016).

19. R. A. Fisher, *Annals of Eugenics* **7**, 355 (1937).

20. A. Kolmogorov, I. Petrovskii, N. Piskunov, *Bull. Moscow Univ. Math. Mech.* **1**, 1–25 (1937).

21. W. van Saarloos, *Physics Reports* **386**, 29 (2003).

22. F. Serwane, *et al.*, *Nature Methods* **14**, 181 (2017).

23. J. J. Williamson, G. Salbreux, *Physical Review Letters* **121**, 238102 (2018).

24. O. Cochet-Escartin, J. Ranft, P. Silberzan, P. Marcq, *Biophysical Journal* **106**, 65 (2014).

25. N. Otsu, *IEEE Transactions on Systems, Man, and Cybernetics* **9**, 62 (1979).

26. F. Family, T. Vicsek, *Journal of Physics A: Mathematical and General* **18**, L75 (1985).

27. A.-L. Stanley, H. E. Barabási, *Fractal Concepts in Surface Growth* (Cambridge University Press, Cambridge, England, 1995).
