# Supplementary material for: Self-propagating wave drives morphogenesis of skull bones in vivo
Source: Nat Commun. 2025 May 9;16:4330. doi: 10.1038/s41467-025-59164-9 (PMC12064835; doi:10.1038/s41467-025-59164-9)
Supplement: Supplementary file 2 — Description of Additional Supplementary Information [file 41467_2025_59164_MOESM2_ESM.pdf]

**Description of Additional Supplementary Files**

File Name: Supplementary Movie 1
Description: *Individual cell borders are complex and dynamic.* Live imaging of mosaic labelling with adenoviral Utr-RFP in wholemount E14.5 skull caps shows complex mesenchymal cell shapes and dynamics.

File Name: Supplementary Movie 2
Description: *Bone expansion ex vivo.* Live imaging of Osx1-GFP::Cre labelled skull caps at E13.75.

File Name: Supplementary Movie 3
Description: *Oriented division are found at the osteogenic front.* Live imaging of E13.75 Osx1-GFP::Cre labelled skull caps.

File Name: Supplementary Movie 4
Description: *Few cellular rearrangements at E13.75.* Tracked nuclei show few neighbor exchanges at osteogenic front in live imaged E13.75 Osx1-GFP::Cre labelled skull caps.

File Name: Supplementary Movie 5
Description: *New differentiation events occur ahead of the osteogenic front.* Live imaging of E13.75 Osx1-GFP::Cre; R26RmTmG reporter mice showing nuclear only label ahead of an osteogenic front labelled with both membrane and nuclear GFP.
